# Learning to Detect Deception from Evasive Answers and Inconsistencies across Repeated Interviews: A Study with Lay Respondents and Police Officers

**DOI:** 10.3389/fpsyg.2017.02207

**Published:** 2018-01-04

**Authors:** Jaume Masip, Carmen Martínez, Iris Blandón-Gitlin, Nuria Sánchez, Carmen Herrero, Izaskun Ibabe

**Affiliations:** ^1^Department of Social Psychology and Anthropology, University of Salamanca, Salamanca, Spain; ^2^Department of Psychology, California State University Fullerton, Fullerton, CA, United States; ^3^Department of Social Psychology and Methodology of the Behavioral Sciences, University of the Basque Country, San Sebastián, Spain

**Keywords:** deception, lie detection, consistency, interviewing, police, deception cues, cognitive load, evasive answers

## Abstract

Previous research has shown that inconsistencies across repeated interviews do not indicate deception because liars deliberately tend to repeat the same story. However, when a strategic interview approach that makes it difficult for liars to use the repeat strategy is used, both consistency and evasive answers differ significantly between truth tellers and liars, and statistical software (binary logistic regression analyses) can reach high classification rates (Masip et al., 2016b). Yet, if the interview procedure is to be used in applied settings the decision process will be made by humans, not statistical software. To address this issue, in the current study, 475 college students (Experiment 1) and 142 police officers (Experiment 2) were instructed to code and use consistency, evasive answers, or a combination or both before judging the veracity of Masip et al.'s (2016b) interview transcripts. Accuracy rates were high (60% to over 90%). Evasive answers yielded higher rates than consistency, and the combination of both these cues produced the highest accuracy rates in identifying both truthful and deceptive statements. Uninstructed participants performed fairly well (around 75% accuracy), apparently because they spontaneously used consistency and evasive answers. The pattern of results was the same among students, all officers, and veteran officers only, and shows that inconsistencies between interviews and evasive answers reveal deception when a strategic interview approach that hinders the repeat strategy is used.

## Introduction

### Between-statement inconsistencies and deception

Humans are poor lie detectors. According to a comprehensive meta-analysis, humans' accuracy in discriminating between truths and lies on the basis of the sender's behavior is 54%, which is just above 50% chance accuracy (Bond and DePaulo, 2006). Discrimination accuracy is poor because (a) communication senders hardly display any behavioral cue to deception (DePaulo et al., 2003; Sporer and Schwandt, 2006, 2007; Hauch et al., 2015); (b) the diagnostic value of deception cues depends on a number of moderator variables (DePaulo et al., 2003; Sporer and Schwandt, 2006, 2007; Hauch et al., 2015); and (c) even the most reliable cues are poorly related to truth or deception (Hartwig and Bond, 2011). These findings suggest that training programs to detect deception on the basis of (fallible) behavioral cues can have only limited success (see Hauch et al., 2016). As suggested by Hartwig and Bond (2011), a more effective approach involves designing interview strategies that elicit or maximize behavioral differences between liars and truth tellers.

Over the last decade, a number of such interview strategies have been developed by behavioral scientists (Vrij et al., 2010; Vrij and Granhag, 2012; for an overview of recent trends in deception research, see Masip, 2017). A promising and forensically relevant approach attempts to elicit between-statement inconsistencies—that is, inconsistencies among separate accounts of the same person (see Vredeveldt et al., 2014). Leins et al. (2011) asked truth tellers and liars to provide verbal and pictorial (drawing a map) descriptions of their whereabouts. The consistency between the verbal statements and the drawings was higher among truth tellers than among liars. Subsequent research showed that the key factor to eliciting inconsistencies in liars was switching the interview mode (from verbal to pictorial or vice-versa; Leins et al., 2012).

Other authors have examined inconsistencies without changing interview format, but manipulating question type instead. Mac Giolla and Granhag (2015), and Granhag et al. (2016) interviewed liars and truth tellers three times using both anticipated questions (i.e., questions that interviewees can reasonably expect to be asked during the interview) and unanticipated questions (i.e., questions that are hardly expected by the interviewees). No difference in consistency was predicted between liars and truth tellers in replying to anticipated questions, but the authors expected liars to be more inconsistent than truth tellers in replying to unanticipated questions. However, the findings did not support their prediction: Liars and truth tellers displayed similar levels of consistency irrespective of question type^1^.

There are at least two possible explanations for the null findings under the unanticipated questions condition. First, the “unanticipated” questions were the same across all interviews; therefore, in reality they were unanticipated only during the first interview. Second, in the studies by Granhag et al. (2016) and Mac Giolla and Granhag (2015), the three interviews “were conducted in direct succession with a minimal waiting period between each interview” (Mac Giolla and Granhag, 2015, p. 145). For inconsistencies to be elicited in liars it is important to increase the retention interval between the interviews: Over time, the memory trace of poorly encoded information, such as imagined rather than perceptually experienced details, or peripheral rather than central details, might become weaker (e.g., Craik and Tulving, 1975). This memory decay can result in omissions during the second or third interview, as well as in contradictions across repeated interviews^2^.

However, other research using long intervals between interviews has also found liars to be about as consistent as truth tellers (Granhag and Strömwall, 2002; Granhag et al., 2003; Strömwall and Granhag, 2005). To explain this evidence, Granhag and Strömwall (1999) proposed the *repeat* vs. *reconstruct hypothesis*. In a deliberate attempt to appear consistent—and hence credible—liars presumably make an effort to repeat the same invented story every time they are interviewed. Conversely, truth tellers simply describe what they recall about the target event. Because memory is reconstructive and error prone (Tulving, 2000; Loftus, 2003), the truth tellers' successive recollections might contain some discrepancies. As a result, the net amount of (in)consistencies displayed by liars and truth tellers is very similar (Granhag and Strömwall, 1999).

There are, however, a number of features in Granhag and Strömwall's studies that might have facilitated the liars' usage of the repeat strategy (see Fisher et al., 2013). First, the participants were aware that they would be interviewed repeatedly. Therefore, they might have rehearsed their statements after the first interview. Research shows that rehearsal can attenuate memory decay (see Dark and Loftus, 1976; Agarwal et al., 2013). Second, all questions were about central aspects of the event; hence, they could have been anticipated by liars (Fisher et al., 2013). Third, the first interview was conducted immediately after the event, the second interview 4 days later, and the final interview 1 week after the second one. The immediate interview and the participants' knowledge that more interviews would be conducted may have inoculated the liars' memory against forgetting. This might have made the liars' memory trace strong even after a 4-day delay, and continued recall attempts of the false stories (especially its core aspects) might have additionally attenuated memory decay (Ebbesen and Rienick, 1998). Fourth, there were no manipulations to make it difficult for liars to invent deceptive responses during the first interview, to experience difficulty encoding their initial responses, or to create demand on retrieval attempts during the subsequent interviews.

A way to hinder fabrication, encoding, and retrieval is by increasing cognitive load. Vrij et al. (2010) listed a number of reasons why during an interview lying is more cognitively taxing than telling the truth. Also, basic cognitive research has shown that lying requires greater access to executive control processes than truth telling (e.g., Debey et al., 2012), and neuroimaging research has revealed that brain areas involved in working memory, response monitoring, response conflict, inhibition, and multitasking are activated to a greater extent during deception than truth telling (Farah et al., 2014; Gamer, 2014; Lisofsky et al., 2014). In short, lying often involves greater cognitive demands than does telling the truth^3^. If the interviewees' cognitive load is artificially increased during the interview, the liars will have fewer resources left compared to truth tellers, and will be less able to effectively cope with the increased cognitive demands. As a result, liars might display more observable signs of cognitive overload (e.g., response latencies, pauses, and a decrease in body movements) than truth tellers.

Supporting these considerations, recent research shows that artificially inducing cognitive load during an interview results in an increase in visible deception cues (Vrij et al., 2016), as well as in observers' ability to more accurately judge veracity (Vrij et al., 2017).

Inducing cognitive load during repeated interviews might generate between-statement inconsistencies in liars. Inventing a deceptive response to an unexpected question on the spot is cognitively demanding; therefore, increasing the liars' cognitive load further during the interview might hinder encoding (e.g., Chandler and Sweller, 1996). Likewise, an increased cognitive load during a subsequent interview might hinder retrieval of poorly encoded responses provided during the first interview (e.g., Craik et al., 1996). These processes can yield inconsistencies between interviews.

### Interviewing strategically to elicit between-statement inconsistencies and evasive answers

Based on the above considerations, Masip et al. (2016b) designed a strategic interview approach to detect false alibies. Guilty participants (*n* = 24) committed a mock crime, while innocent participants (*n* = 24) performed four tasks under the guidance of an experimenter. Both guilty and innocent participants were subsequently informed that they were suspects of the crime and would be interviewed. Their task was to convince the interviewer that they were innocent and had performed the innocent participants' activities. To prepare their alibi, guilty participants were given the opportunity to “search for information” by requesting from the experimenter all details about the innocent tasks that they deemed necessary to convince the interviewer of their innocence.

The interview was conducted immediately, and was repeated *unexpectedly* after a 1-week retention interval. It focused on the alibi (i.e., the activities of the innocent participants), and contained eight *central questions* (questions about the actions performed by the innocent participants and about the core details; these details cannot be changed without changing the story), and eight *peripheral questions* (questions about details and actions that were secondary to the event; these were aspects that could be altered with the central storyline remaining unchanged). An example of a central question is “What was the first task?”; an example of a peripheral question is “How many chairs were in the office room?” (see Appendix 1 in Supplementary Material for the full set of relevant questions; Questions 4, 6, 7, 9, 10, 13, 15, and 16 were central, while Questions 3, 5, 8, 11, 12, 14, 17, and 18 were peripheral). All questions were about specific details, thus requiring short answers; this would facilitate the measurement and coding of the dependent variables.

The interviewees' cognitive load was increased during the interview by asking them to reply to all questions and to do so as soon as possible after each question. Specifically, the interviewees were told that delayed responses could indicate deception, and the interviewer held a chronometer through the entire interview. Asking to reply quickly is cognitively demanding because retrieving information from long term memory requires time, particularly if the memory is poorly encoded—as was probably the case among the liars in Masip et al.'s (2016b) study, who did not perform the innocent tasks but merely learned about them from the experimenter. Further, a guilty suspect who ignores or cannot retrieve the relevant information needs to fabricate a plausible answer on the spot to avoid detection; this task can be very demanding (see Vrij et al., 2010; Walczyk et al., 2014), requiring time and concentration. Because of these reasons, the need to reply quickly might be extremely taxing for liars.

Note that Masip et al. (2016b) took measures to hinder the liars' “repeat strategy”: The participants were unaware that they would be interviewed again, the time between the first and the second interview was relatively long, peripheral questions were asked in addition to central questions, and cognitive load was induced. The authors found that, as predicted, guilty suspects requested central rather than peripheral information from the experimenter to prepare their alibi. This finding suggested that liars would have more information about the central aspects of the innocents' tasks than about the peripheral aspects. The main dependent measures were *response accuracy, consistency across interviews*, and *evasive answers*. If the suspect gave the same answer to the same question in both interviews, that was coded as a consistent response. If the suspect gave semantically different answers, that was coded as an inconsistent response. Evasive answers were replies that contained no information, such as saying “I don't remember,” or replying “there was no poster” when asked on which wall there was a poster. The rationale behind measuring evasive answers was that guilty participants urged to reply quickly can resort to answers of this kind whenever they can neither retrieve the correct answer nor invent a plausible one.

Masip et al. (2016b; see also Blandón-Gitlin et al., 2017) predicted that, relative to truth tellers, liars would score lower on response accuracy and consistency, and higher on evasive answers. These hypotheses were supported by the data. The authors also predicted that the differences between liars and truth tellers in terms of the three dependent measures would be larger in responding to peripheral than to central questions. However, for response accuracy the difference was of the same magnitude regardless of centrality, for consistency it was larger for responses to central questions than for responses to peripheral questions^4^, and for evasive answers it was larger for responses to peripheral than to central questions, as expected—though, importantly, the difference in evasive answers between liars and truth tellers was significant for both responses to peripheral questions and responses to central questions (for more detail about the predictions and the interpretation of the results, see Masip et al., 2016b).

Note that consistency was found to be an indicator of truthfulness, while evasive answers were found to signal deception. However, whenever a participant gave the same evasive answer (e.g., “I don't remember”) in response to the same question in both interviews, that was coded as a consistent reply (= a truth indicator), even though evasive answers were found to indicate deception, not truthfulness. To address this issue, Masip et al. (2016b) created a new variable that combined consistency and evasive answers. Specifically, inconsistencies were coded as 0, consistencies due to a repeated evasive answer were also coded as 0, and all other consistencies were coded as 1. In this way, 0 always denoted deception and 1 always denoted truthfulness. As expected, scores on this variable were significantly higher for innocent than for guilty suspects. The Guilt Status × Question Type (central vs. peripheral) interaction was not significant, indicating that the difference between innocent and guilty participants was similar irrespective of question centrality.

Masip et al. (2016b) conducted binary logistic regression analyses (BLRAs) with the leave-one-out cross validation method to see how well truthful and deceptive interviews could be detected on the basis of inconsistencies and evasive answers. Classification rates were 69% on the basis of consistency (71% for truths and 67% for lies), 73% on the basis of consistency for central questions (87% for truths and 58% for lies), 87% (for both truths and lies) on the basis of evasive answers, and 94% on the basis of the combined consistency and evasive answers variable (96% for truths and 92% for lies). The interviewers in Masip et al.'s (2016b) study also judged veracity. There were 22 interviewers who questioned an average of 4.36 suspects each (conducting either the first or the second interview, but not both, for each of the suspects they questioned). Each interviewer made a dichotomous veracity judgment in a form immediately after questioning each suspect. The interviewers' mean accuracy rate was 54% (71% for truths and 40% for lies). Clearly, the BLRAs classification rates compare well to the interviewers' accuracy rates.

### Current study

Masip et al.'s (2016b) BLRAs classification rates were fairly high, but they were derived from statistical analyses performed by a computer program. If the interview procedure is ever to be used in real life, the decision process will be made by humans, not computers. Therefore, it is critical to examine whether human beings instructed to use the diagnostic cues identified by Masip et al. (2016b) perform well enough in classifying the interviews as truthful or deceptive. In particular, we were interested in knowing whether humans would be able to reach accuracy rates comparable to the classification rates of the BLRAs conducted by Masip et al. (2016b).

The purpose of the current study was to address this issue. Human participants read the transcripts of Masip et al.'s (2016b) interviews (each individual participant read only a subset of the interviews) and indicated whether each suspect lied or told the truth. Recall that in the study by Masip et al. (2016b), consistency across peripheral and central questions, consistency in responding to central questions, evasive answers, and the combination (in the specific way explained above) of consistency and evasive answers significantly discriminated between guilty suspects (liars) and innocent suspects (truth tellers). These cues also resulted in high BLRAs classification rates. Therefore, we instructed four groups of participants to judge the veracity of suspects by using either consistency (consistency condition), consistency in answering to central questions (consistency-central condition), evasive answers^5^ (evasive-A condition), or the combined consistency and evasive answers variable (consistency-evasive condition). We also included two control groups in the design: A no-instruction group (uninstructed condition) whose performance was expected to be close to chance accuracy, and a so-called “information group” (information condition) who was informed about the correct answers to all interview questions and thus was expected to perform close to perfection. In Experiment 1, college students acted as participants. In an attempt to increase ecological validity, we ran Experiment 2 with law enforcement officers as participants. For both experiments, we predicted that instructed participants would be fairly able to discriminate between truthful and deceptive statements, performing better than participants in the uninstructed control condition.

## Experiment 1

### Method

#### Participants

The participants were 475 psychology students (386 females and 89 males; *M* age = 19 years; *SD* = 2.82). They participated on a voluntary basis but were incentivized with an academic reward (a slight increase in the course grade).

#### Materials

All participants received a set of interview transcripts and a response sheet, along with detailed written instructions. Following the instructions, the participants in the experimental conditions had to mark each consistent reply, evasive answer, etc. on the transcript pages. They also had to calculate the percentages (of consistent replies, evasive answers, etc.) for each suspect, write the figures on the response sheet, and make a dichotomous true/lie decision for each suspect. A more detailed description of the materials and procedures follows.

##### Interview transcripts

Masip et al. (2016b) collected data from 24 truth tellers and 24 liars. All 48 participants were interviewed twice. Therefore, we had 48 pairs of interviews—one pair from each suspect. In order to avoid tiredness and boredom among raters, as well as to allow them to do the rating task within a reasonable time, we limited the number of interview pairs (or suspects) to be rated by each participant to 12. Therefore, we divided the 48 interview pairs in four sets. Each set contained six truthful and six deceptive pairs. We ensured that the suspects' gender and age did not differ significantly across truths and lies and interview sets. We additionally created two versions of each set by counter-balancing the presentation order of the interview pairs. Specifically, the order of the “direct” (D) version of the set was determined randomly, and then it was reversed for the “reverse” (R) version of the set, such that interview pair (or suspect) number 1 in the D version was number 12 in the R version, pair number 2 in the D version was number 11 in the R version, and so forth.

The transcripts of the interview pairs were printed and arranged in 12-page booklets, each page containing one interview pair. There were separate booklets for Set 1-D (direct), Set 1-R (reverse), Set 2-D, Set 2-R, Set 3-D, Set 3-R, Set 4-D, and Set 4-R. As shown in Appendix 1 (Supplementary Material) (transcript page for the consistency condition corresponding to Suspect 7 in Set 3-D), each page in each booklet contained the set number (1 through 4) and order (D or R), the participant (i.e., suspect) number (1 through 12), a column with all interview questions, a column with the suspect's answers to each question during the first interview, and another column with the suspect's answers to each question during the second interview. Except for the uninstructed control group, whose participants had to read the transcripts and make their veracity judgments intuitively, additional columns were added such that the raters could calculate the percentage of consistencies (consistency condition), evasive answers (evasive-A condition), etc. for each suspect. For example, as shown in Appendix 1 (Supplementary Material), for the consistency condition, two additional columns were added. For those questions to which the suspect had given the same answer in the two interviews, the raters had to write “1” in the “Same answer? YES” column. For those questions to which the suspect had given a different answer in each interview, the raters had to write “1” in the “Same answer? NO” column. Then, to calculate the percentage of consistent answers out of 16, the raters had to sum the numbers of the “Same answer? YES” column and multiply the result times 6.25^6^. Finally, they had to write the result in the final cell on the page. Similar operations had to be done by the other experimental groups.

##### Response sheet

The response sheet was used to collect the raters' demographic information (age and gender) as well as their veracity judgments. Specifically, the raters had to compare the final percentage they had calculated for each suspect (see previous paragraph) with a specific cutoff score taken from the BLRAs of Masip et al.'s (2016b) study to determine whether each individual suspect was truthful or deceptive^7^. They had to express their judgments on the response sheet.

The uninstructed participants had to make their judgments using their own intuitive criteria; therefore, their response sheet was slightly different in that they had to briefly report in writing the reasons for their truth and lie judgments.

##### Correct answers sheet

The information condition participants had to code whether each answer given to each question in each interview was correct or not. To do this task, they received a sheet with all 16 critical questions and the correct answer to each question.

##### Instructions

Detailed instructions were written for each group. The instructions contained some basic information about the setup of Masip et al.'s (2016b) study, as well as detailed guidelines on how to proceed to complete the final columns on the transcript pages, how to make all of the calculations, and how to fill in the final response sheet. For all the instructed conditions, the need to closely follow the instructions and not to make intuitive judgments or use cues other than the ones described in these instructions was emphasized^8^.

#### Procedure

The sessions were run during a regular Social Psychology lecture. Each class was assigned to a different condition (the allocation of students to a specific class is made by the School administration on the basis of the first letter of the students' surnames). The number of participants in each condition is displayed in the first row in Table 1. To avoid social interactions during the sessions, all students were sat leaving an empty seat on each side. The students were informed that participation was voluntary, and that they could withdraw at any time. After signing a consent form, they received the written instructions, the booklets with the transcripts^9^, and the response sheets. The information condition participants also received the correct answers sheet.

**Table 1:** Sample size, mean accuracy rates, and standard deviations for the separate conditions in Experiment 1.

**Measure**	**Consistency**	**Consistency-central**	**Uninstructed**	**Evasive-A**	**Consistency-evasive**	**Information**
*n*	79	74	81	77	76	88
*M*	64.51^a^	73.65^b^	75.93^b^	86.97^c^	87.83^c^	98.77^d^
*SD*	9.66	10.60	15.48	5.29	11.66	2.97

*The conditions are sorted according to mean accuracy rates. Scheffé tests were conducted for pairwise comparisons; within each row, means with different superscripts differ significantly from each other*.

One experimenter carefully read aloud and clarified the instructions. Next, the participants worked on the task on their own. After all participants had finished, the experimenters collected all materials and debriefed the participants.

#### Coding

Uninstructed participants (*n* = 81) indicated the reasons behind their veracity judgments. In all, they provided 193 reasons for their truth judgments, and 254 reasons for their lie judgments. A researcher went over all responses and, using a data-driven (bottom-up) approach, created a coding scheme with a number of individual cues arranged in cue categories (Table 2). He also wrote a five-page booklet with the descriptions of all cues. Two research assistants blind to the hypotheses read the booklet and independently coded all individual responses. Reliability (Cohen's Kappa) is displayed in Table 2. Inter-rater discrepancies were resolved by discussion.

**Table 2:** Reasons provided by the uninstructed participants in Experiment 1 to explain their truth and lie judgments.

**Cue categories and individual cues**	**Kappa**	**Total percentage**	**Truth judgments (percentage)**	**Lie judgments (percentage)**	**Significance of McNemar test**
**CONSISTENCY/INCONSISTENCY ACROSS INTERVIEWS**	**0.93**	**90.12**	**76.54**	**82.72**	**0.332**
Consistency across interviews	0.96	66.67	59.26	12.35	<0.001
Inconsistency across interviews	0.89	51.85	0.00	51.85	<0.001
Better recall during Interview 1	0.96	20.99	20.99	0.00	<0.001
Better recall during Interview 2	0.98	30.86	2.47	28.40	<0.001
Unspecific	0.65	4.94	4.94	2.47	0.500
**CONFIDENCE/DOUBTS AND LACK OF CONFIDENCE**	**0.96**	**64.20**	**35.80**	**48.15**	**0.134**
Confidence or certainty	0.87	29.63	24.69	8.64	0.007
Doubts or lack of confidence	0.87	51.85	11.11	41.98	<0.001
Unspecific	0.32	2.47	2.47	1.23	1.000
**DETAILS OR KNOWLEDGE**	**0.89**	**58.02**	**30.86**	**53.09**	**0.001**
Detailed or precise answers	0.88	30.86	19.75	11.11	0.230
Vague or unspecific answers	0.83	23.46	4.94	18.52	0.019
Ignoring the answers	0.92	34.57	3.70	30.86	<0.001
Unspecific	0.91	6.17	2.47	3.70	1.000
**CLARITY AND CONCISION**	**0.85**	**34.57**	**24.69**	**20.99**	**0.648**
Clear and/or concise answers	0.95	27.16	19.75	8.64	0.078
Ambiguous or unclear reply, verbiage…	0.72	17.28	3.70	13.58	0.057
Unspecific	0.39	2.47	2.47	0.00	0.500
**ATTITUDE**	**0.88**	**25.93**	**16.05**	**16.05**	**1.000**
Spontaneity	0.89	13.58	13.58	0.00	0.001
Nervousness or lack of spontaneity	0.83	14.81	0.00	14.81	<0.001
Unspecific	0.80	2.47	2.47	1.23	1.000
**UNDEFINED CONSISTENCY/INCONSISTENCY**	**0.78**	**17.28**	**7.41**	**13.58**	**0.227**
Undefined consistency	0.69	7.41	6.17	2.47	0.375
Undefined inconsistency	0.75	13.58	3.70	11.11	0.109
**CONSISTENCY/INCONSISTENCY AMONG INTERVIEWEES**	**0.87**	**14.81**	**9.88**	**9.88**	**1.000**
Consistency among interviewees	0.76	8.64	4.94	3.70	1.000
Inconsistency among interviewees	0.91	6.17	1.23	4.94	0.375
Unspecific	–	3.70	3.70	1.23	0.500
**CONSISTENCY/INCONSISTENCY WITHIN AN INTERVIEW**	**0.53**	**7.41**	**4.94**	**3.70**	**1.000**
Consistent responses	−0.01	4.94	4.94	0.00	0.125
Inconsistent responses	0.85	3.70	0.00	3.70	0.250
Unspecific	–	0.00	0.00	0.00	−
**Other**	**0.57**	**7.41**	**6.17**	**3.70**	**0.625**

### Results

Examination of the transcript pages and response sheets revealed that some participants had inadvertedly committed some errors (such as leaving a row blank in a transcript page, making calculation errors in summing or multiplying numbers in the transcript pages, etc.). These errors were corrected and the analyses were run both on the uncorrected and on the corrected data. Because in real life coders can also make the same kind of errors made by our participants, and because we did not want to manipulate the participants' responses, the results reported in the current paper were obtained with the uncorrected data set. If, as suggested by a reviewer, in real life practitioners presumably commit fewer errors than research participants because they are more careful and double-check the numbers, then the current results represent conservative estimates of the potential of the proposed interview approach and raters' instruction. However, for the sake of transparency, all of the analyses and descriptive statistics provided here for the uncorrected data are also provided in Appendix 2 (Supplementary Material) with the corrected data. Comparison of both sets of results reveals that they are virtually identical. Indeed, because the errors only changed the percentages slightly, they had a minimal impact on the final veracity judgments. This paragraph also applies to Experiment 2.

#### Accuracy

A mixed 2 (Veracity) x 6 (Condition: Consistency, Consistency-Central, Evasive-A, Consistency-Evasive, Control, and Information) ANOVA, with repeated measures on the veracity factor, was conducted on accuracy (percentage of correct lie/truth judgments). The veracity main effect was significant, *F*_(1, 469)_ = 4.70, *p* = 0.031, $\eta_{\text{p}}^{2}$ = 0.010, indicating that truths (*M* = 82.68, *SD* = 19.55) were slightly better identified than lies (*M* = 80.52, *SD* = 19.28). The condition main effect was also significant, *F*_(5, 469)_ = 120.74, *p* < 0.001, $\eta_{\text{p}}^{2}$ = 0.563. Descriptive data are displayed in Table 1. The lowest accuracy rate corresponded to the consistency condition, *M* = 64.51, *SD* = 9.66, though it was nevertheless rather substantial—note that chance accuracy was 50%. Unexpectedly, the uninstructed group performed at the same level as the consistency-central group, judging correctly as liars or truth tellers roughly three out of every four suspects. Finally, the two groups using evasive answers (Evasive-A and Consistency-Evasive) performed at the same level, close to 90% accuracy (Table 1).

**Figure 1:**
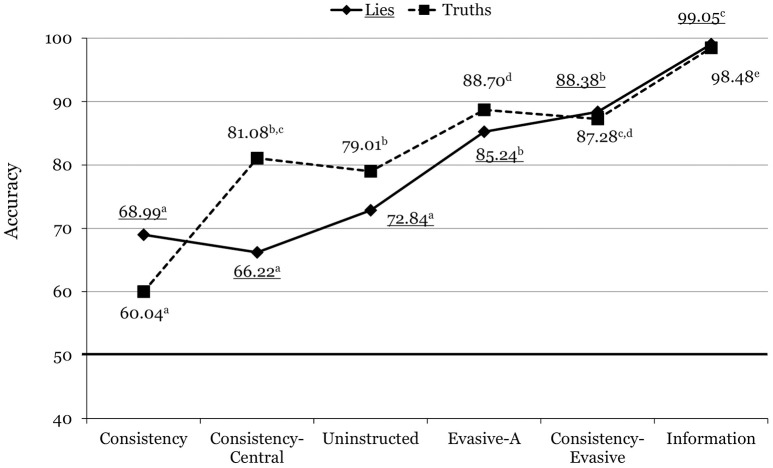
Mean accuracy rates across the different conditions in Experiment 1. For each line, means with different superscripts differ significantly from each other. Underlined values correspond to lies.

The Veracity x Condition interaction was also significant, *F*_(5, 469)_ = 9.10, *p* < 0.001, $\eta_{\text{p}}^{2}$ = 0.088. As shown in Figure 1, accuracy for truths increased more abruptly across the conditions than accuracy for lies (which did not vary significantly across the consistency, consistency-central, and uninstructed conditions), though the two lines ultimately converge. Bonferroni-adjusted pairwise comparisons revealed that whereas there were significant differences between the identification of truths and lies in the consistency (*p* = 0.001), consistency-central (*p* < 0.001), and uninstructed (*p* = 0.017) conditions, the difference was not significant for the evasive-A (*p* = 0.192), consistency-evasive (*p* = 0.681), and information (*p* = 0.819) conditions. Thus, not only were the two groups using evasive answers more accurate overall than the uninstructed group and the two consistency groups, but also less biased toward making truth or lie judgments.

#### Cues used by the uninstructed raters

Examination of the reasons given by uninstructed control participants for their judgments (Table 2) can help clarify why their accuracy was so high. Here we focus on the cue categories mentioned by at least 25% of respondents. These cue categories were consistency/inconsistency across interviews (mentioned by 90% of respondents; see third column in Table 2), confidence vs. doubts and lack of confidence (64%), details or knowledge (58%), clarity and concision (35%) and attitude (26%). It is apparent from the fourth and fifth columns in Table 2 that 59.26% of uninstructed respondents mentioned consistency across interviews as a reason for their truth judgments, whereas only 12.35% of them mentioned this cue as a reason for their lie judgments. The difference was significant (see final column in Table 2).

Further examination of Table 2 reveals that in addition to consistency across interviews, better recall during the first than during the second interview, showing confidence or certainty, and displaying spontaneity in the responses were also mentioned significantly more often to justify truth than lie judgments. On the other hand, inconsistencies across the two interviews, better recall during the second than during the first interview, doubts or lack of confidence, giving vague or unspecific details, ignoring the answers, and showing nervousness or lack of spontaneity were mentioned most often as a justification for deception judgments. The responses being clear and/or concise was mentioned marginally more often to justify truth than lie judgments, and ambiguous, unclear, or verbose replies were mentioned marginally more often to justify lie rather than truth judgments (Table 2).

It is apparent from these data that the uninstructed participants spontaneously used some valid cues. Specifically, 90% of them spontaneously used (in)consistencies across the two interviews, and 35% of them used evasive answers (the raters' category “ignoring the answers” largely overlaps with our “evasive answers” lie indicator).

### Discussion

After coding consistency and/or evasive answers by themselves, humans were able to classify the truthful and deceptive interviews as accurately as the BLRAs conducted by Masip et al. (2016b). The only exception were the participants in the consistency-evasive condition, whose accuracy rates (around 88%) were slightly lower than the BLRAs classification rates using the same combined consistency and evasive answers cue (about 94%).

The current results indicate that consistency can become an indicator of veracity that can be used by human detectors, provided a specific kind of interview is used that makes it difficult for liars to employ the repeat strategy. However, evasive answers allow for a better discrimination between truths and lies than consistency, with accuracy rates approaching 90%.

An advantage of the current interviewing approach is that even the uninstructed raters picked on the discriminative cues and used them correctly, attaining fairly high accuracy rates (about 75%).

## Experiment 2

The participants in Experiment 1 were college students. However, the current interview procedure was designed to discriminate between truthful and deceptive alibies in forensic contexts. In Experiment 2, we examined the effectiveness of instructing law enforcement officers to code consistency, evasive answers, and the combined variable, as well as to identify the truthful and deceptive interviews on the basis of these cues. We used the same instruction and control conditions as in Experiment 1.

### Method

#### Participants

The participants were 142 police officers (26 females and 116 males; *M* age = 40 years; *SD* = 8.16; age range: 25–55) who were studying at the National Police School in Spain to become police inspectors. On average, they had 15 years of job experience, *SD* = 9.62.

#### Procedure and materials

The data were collected at the Spanish National Police School. As in Experiment 1, each class was assigned to a separate condition (the officers' allocation to a specific class is based on the first letter of their surnames). The number of participants in each condition is displayed in the top panel of Table 3. The participants signed an informed consent form. The procedures and materials used were identical to Experiment 1, except that because the number of available officers was (comparatively) so small (*N* = 142) we used only one set of transcripts—specifically Set 2, which was the most representative of the results across all four sets. Also, questions were added to the response sheet to record whether each police respondent was novice or experienced, as well as the specific length (in years) of their job experience.

**Table 3:** Sample size, mean accuracy rates, and standard deviations for the separate conditions in Experiment 2.

**Measure**	**Consistency**	**Consistency-central**	**Uninstructed**	**Evasive-A**	**Consistency-evasive**	**Information**
**ALL OFFICERS**						
*n*	24	25	22	26	22	23
*M*	59.72^a^	71.53^b^	76.89^b,c^	85.45^c,d^	93.18^d,e^	98.91^e^
*SD*	10.33	9.72	14.30	8.06	9.50	5.21
**VETERAN OFFICERS ONLY**						
*n*	12	16	16	17	17	15
*M*	61.11^a^	70.10^a^	73.44^a,b^	84.22^b^	91.67^b,c^	100.00^c^
*SD*	12.97	11.06	13.68	8.29	10.21	0.00

*The conditions are sorted according to mean accuracy rates. Scheffé tests were conducted for pairwise comparisons; within each row, means with different superscripts differ significantly from each other*.

#### Coding

The 22 respondents in the uninstructed control condition provided 36 reasons for their truth judgments and 50 reasons for their lie judgments. Two naïve coders were trained to use the coding scheme developed in Experiment 1. However, the relatively small number of participants in Experiment 2 (only 22 in the uninstructed condition compared to 81 in that condition in Experiment 1) and the consequent lower frequency of reasons for Experiment 2 were problematic. For instance, some cues were rated 0 (cue absent) for all senders by one (or even by the two) coder(s). In addition, the coders had difficulty in differentiating between vague or unspecific answers and ignoring the answers; therefore, we merged these two cues and labeled the resulting cue as “lack of knowledge.” Table 4 contains the reliability (Kappa coefficients) and other statistics for the individual cues mentioned by at least 25% of the uninstructed participants. Discrepancies between the raters were resolved by discussion.

**Table 4:** Most frequent reasons provided by the uninstructed participants in Experiment 2 to explain their truth and lie judgments.

**Cue categories and individual cues**	**Kappa**	**Total percentage**	**Truth Judgments (percentage)**	**Lie judgments (percentage)**	**Significance of McNemar test**
**CONSISTENCY/INCONSISTENCY ACROSS INTERVIEWS**					
Consistency across interviews	0.94	45.45	40.91	4.55	0.021
Inconsistency across interviews	0.85	36.36	0.00	36.36	0.008
**CONFIDENCE/DOUBTS AND LACK OF CONFIDENCE**					
Doubts or lack of confidence	1.00	27.27	0.00	27.27	0.031
**DETAILS OR KNOWLEDGE**					
Detailed or precise answers	0.91	31.82	31.82	0.00	0.016
Lack of knowledge	0.62	40.91	9.09	36.36	0.070
**UNDEFINED CONSISTENCY/INCONSISTENCY**					
Undefined consistency	0.81	31.82	31.82	0.00	0.016
Undefined inconsistency	0.81	27.27	0.00	27.27	0.031

### Results and discussion

#### Accuracy

We conducted a mixed 2 (Veracity) × 6 (Condition) ANOVA, with repeated measures on the veracity factor, on accuracy. The veracity main effect was not significant, *F*_(1, 136)_ = 2.20, *p* = 0.141, $\eta_{\text{p}}^{2}$ = 0.016. A significant main effect for condition revealed that accuracy varied across conditions, *F*_(5, 136)_ = 60.00, *p* < 0.001, $\eta_{\text{p}}^{2}$ = 0.652. As shown in the top panel of Table 3, the findings resembled those of Experiment 1 in that the sorting of the conditions in terms of accuracy rates was exactly the same. The uninstructed group performed again fairly well (its accuracy rate did not differ significantly from either the consistency-central or the evasive-A condition). Importantly, the consistency-evasive condition accuracy was over 90% and did not differ significantly from the accuracy of the information group. In other words, the combination of consistency and evasive answers (consistency-evasive condition) allowed for similar accuracy rates in detecting truths and lies as having direct access to the truth (information condition; see Table 3).

**Figure 2:**
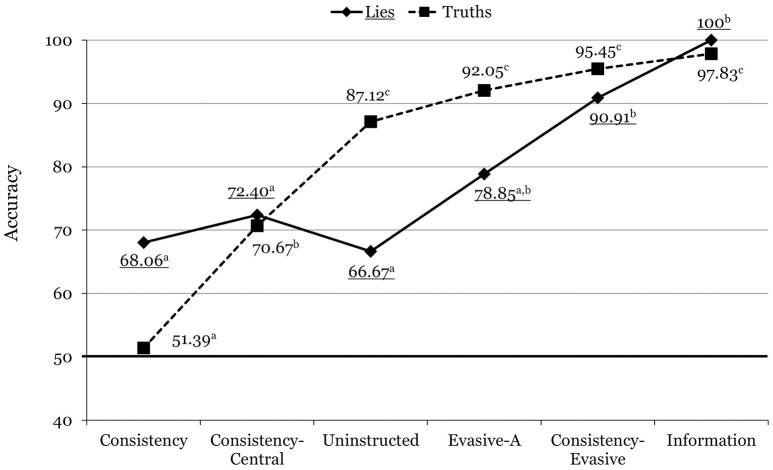
Mean accuracy rates across the different conditions in Experiment 2. For each line, means with different superscripts differ significantly from each other. Underlined values correspond to lies.

The Veracity x Condition interaction was also significant, *F*_(5, 136)_ = 7.16, *p* < 0.001, $\eta_{\text{p}}^{2}$ = 0.208. As shown in Figure 2, the pattern of results was similar to that of Experiment 1 in that the increase in accuracy across the conditions was more abrupt for truths than it was for lies (again, for lies there were no significant differences among the consistency, the consistency-central, and the evasive-A conditions). Bonferroni-adjusted pairwise comparisons revealed that in Experiment 2, the evasive-A participants detected truths significantly better than lies (*p* = 0.005), but the difference was not significant for consistency-evasive participants (*p* = 0.368). Further, accuracy for the consistency-evasive condition did not differ from accuracy for the information condition for either truths or lies (Figure 2).

#### Cues used by the uninstructed raters

As shown in Table 4, the uninstructed condition officers indicated they made truth judgments when they perceived the answers to be consistent across the two interviews, detailed or precise, and presenting “undefined consistency”^10^. Also, officers indicated they made lie judgments when they perceived inconsistencies across the two interviews, doubts or little confidence, that the suspect had little knowledge (*p* = 0.070), and “undefined inconsistency.” The findings indicate that just like the uninstructed students, uninstructed officers used some valid cues (consistency/inconsistency across interviews and, to some extent, lack of knowledge–evasive answers) to make their judgments.

#### Experienced officers

In general, experienced officers differ from novice officers. For instance, relative to novice officers, experienced officers have stronger beliefs about stereotypical deception cues, make their deception judgments with more confidence, and are dispositionally more predisposed toward questioning the veracity of the messages produced by others (Masip and Garrido, 2001; Masip et al., 2005, 2016a; Hurst and Oswald, 2012). Therefore, the question remains whether experienced and novice officers can benefit to the same extent from Masip et al.'s (2016b) interview procedure and instruction.

While some of the officers who participated in the current experiment where novice (*n* = 49), having spent < 2 years in the police force, most were veteran (*n* = 93; 10 females and 83 males, *M* age = 45 years; *SD* = 4.36; age range = 36 to 55) and had an average job experience of 22 years (*SD* = 4.20, range = 15 to 34). Unfortunately, the small number of available novice officers prevented us from making formal comparisons between novice and veteran officers; however, we did conduct analyses including only the seasoned officers to see what the results looked like. The number of experienced officers in each condition is displayed in the lower panel of Table 3.

**Figure 3:**
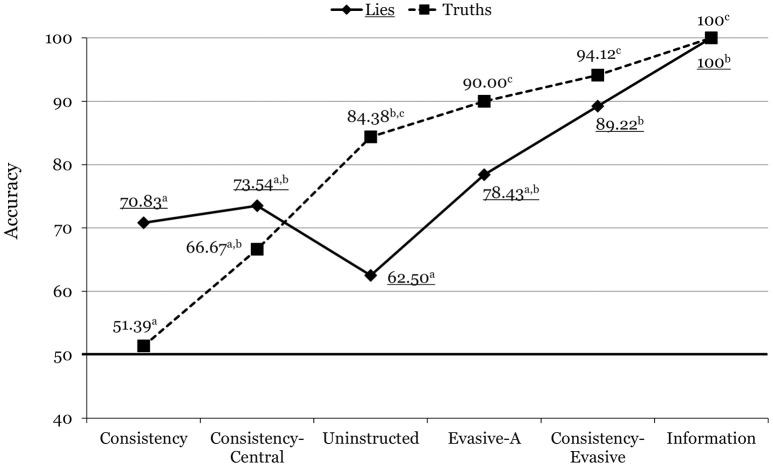
Mean accuracy rates across the different conditions for experienced officers in Experiment 2. For each line, means with different superscripts differ significantly from each other. Underlined values correspond to lies.

A 2 (Veracity) × 6 (Condition) ANOVA yielded a significant main effect for condition, *F*_(5, 87)_ = 28.34, *p* < 0.001, $\eta_{\text{p}}^{2}$ = 0.620, showing that veteran officers can benefit from our interview format and instruction (Table 3). The Veracity x Condition interaction was also significant, *F*_(5, 87)_ = 4.89, *p* = 0.001, $\eta_{\text{p}}^{2}$ = 0.219, producing a pattern of results which was very similar to those of the students and of all officers combined (see Figure 3).

#### Comparing officers and non-officers

To examine whether officers and students benefited to the same extent from the interview format and the different instruction conditions, we conducted a direct comparison between the officers and those students from Experiment 1 who had rated the same transcript set as the officers (for these students, *n* = 116; 20 were in the consistency condition, 19 in the consistency-central condition, 18 in the evasive-A condition, 19 in the consistency-evasive condition, 19 in the uninstructed control condition, and 21 in the information condition). Specifically, we conducted a Sample (students vs. officers) × Veracity × Condition ANOVA. Neither the sample main effect, *F*_(1, 246)_ = 2.28, *p* = 0.132, $\eta_{\text{p}}^{2}$ = 0.009, nor any of the interactions involving sample were significant, all *F*s ≤ 1.22, all *p*s ≥ 0.301. The only significant effects were (again) the condition main effect, *F*_(5, 246)_ = 102.99, *p* < 0.001, $\eta_{\text{p}}^{2}$ = 0.677, and the Veracity x Condition interaction, *F*_(5, 246)_ = 9.41, *p* < 0.001, $\eta_{\text{p}}^{2}$ = 0.161. In short, the beneficial effects of our interview procedure and instruction conditions were the same among police officers and students.

## General discussion

Recent trends in deception detection research focus on designing strategic interview approaches to elicit verbal and nonverbal differences between truth tellers and liars. Some of these approaches have attempted to elicit between-statement inconsistencies, and have been successful only by changing the interview mode (Leins et al., 2011, 2012), but not keeping interview mode constant (Mac Giolla and Granhag, 2015; Granhag et al., 2016). It has been suggested that while liars make a conscious effort to repeat the same story throughout the interviews, which enhances consistency, truth tellers simply try to retrieve their memory for the target event every time they are questioned. However, the fallibility of memory (e.g., Tulving, 2000; Loftus, 2003) impairs the consistency of the truth tellers' accounts. As a result, the degree of inconsistency is similar among truth tellers and liars (Granhag and Strömwall, 1999; Vredeveldt et al., 2014).

Masip et al. (2016b) proposed that inconsistencies can be elicited in liars with strategic interviewing if certain measures are taken to prevent liars to use a “repeat strategy.” Based on the way human memory works, they designed an interview approach where measures were taken to limit the liars' use of the “repeat strategy.” As a result, truths and lies were classified accurately about 70% of the time using consistency as the only predictor in a BLRA. The authors also examined evasive answers, which permitted 87% classification accuracy. Finally, a combination of consistency and evasive answers resulted in classification rates above 90%.

A weakness of Masip et al.'s (2016b) study is that the classifications were derived from computerized statistical analyses rather than being done by humans. In the current study, college students (Experiment 1), and police officers (Experiment 2) read the transcripts, tallied the number of consistencies and/or evasive answers, calculated their percentages, and made truth/lie judgments. A no-instruction control group and an informed group were included in the design for comparison purposes. The findings revealed that, in line with our prediction, both students and officers were able to attain high levels of detection accuracy. Furthermore, the pattern of results was strikingly similar across students, all officers, and experienced officers only. The fact that the groups differed not only in occupation and length of job experience, but also in terms of gender and age attests to the robustness of the findings.

### Overall accuracy

Across all samples, the ordering of the conditions from lower to higher overall accuracy (i.e., accuracy across truths and lies) was consistency, consistency for central questions, uninstructed, evasive answers, combination of consistency and evasive answers, and informed (see Tables 1, 3). Thus, even though consistency across both central and peripheral questions allowed for accuracy rates around 60% (see the consistency condition column in Tables 1, 3), all other conditions permitted better accuracy rates. Evasive answers were indeed more useful than consistencies (accuracy for the evasive-A and the consistency-evasive conditions was always significantly higher than accuracy for the consistency and the consistency-central conditions; see Tables 1, 3), and the combination of consistency and evasive answers resulted in accuracy rates slightly (non-significantly) higher than just evasive answers and, in Experiment 2, not significantly different from the almost-perfect accuracy rates of the informed group^11^. Overall accuracy rates for the consistency-central and the evasive-A conditions were similar to the classification rates of the BLRAs conducted by Masip et al. (2016b), while humans' accuracy for the consistency and the consistency-evasive conditions was somewhat lower than the computerized classifications (but still around 90% for this latter condition).

### Accuracy for truths and lies

Examination of the Veracity x Condition interactions (Figures 1–3) offers more nuanced conclusions. First, apparently, the comparatively modest overall accuracy for the consistency condition was due to a poor identification rate for truths. Put another way, participants in the consistency condition did reasonably well (close to 70%) in identifying lies, but much worse in identifying truths. This finding departs from Masip et al.'s (2016b) computerized classification rates (67% for lies, and 71% for truths), and suggests that the respondents detected many inconsistencies not only in judging the deceptive interviews, but also in judging the truthful ones.

Second, the accurate detection of truthful interviews was much higher for the consistency-central compared to the consistency condition, which indicates that those inconsistencies in truthful interviews that limited the consistency condition accuracy were found in the responses to peripheral rather than to central questions. This makes sense, since honest suspects could have forgotten many secondary details over the 1-week retention interval between the first and the second interview. However, if this were the case, it is unclear why the two coders in Masip et al.'s (2016b) study did not find so many inconsistencies in the truthful suspects' responses to peripheral questions. Apparently, raters in the consistency condition of the current study underestimated the degree of consistency in the responses to the peripheral questions, which led them to display a lie bias. The reasons why this happened are unclear.

Third, whereas accuracy for truths increased progressively across all conditions, accuracy for lies did not change across the consistency, consistency-central, and uninstructed conditions. Similar to Masip et al.'s (2016b) classification rates, consistency for central questions (relative to consistency across both question types) increased the correct identification of truths but not of lies. Thus, apparently, while consistency for central questions is a strong indicator of honesty, inconsistency for central questions is not such a strong indicator of deception.

Fourth, accuracy for truths was very high for the evasive-A and the consistency-evasive conditions, and accuracy for lies increased steadily from the uninstructed condition through the consistency-evasive condition. Fifth, as a result, the consistency-evasive condition accuracy rate was similar for truths and lies, and was the highest among all the groups except the informed one. In conclusion, the combination of consistency and evasive answers (following the coding procedure described in the introduction) permits the highest discrimination (about 90%) of both truths and lies using Masip et al.'s (2016b) interview procedure.

### Uninstructed control group

Contrary to our expectations, the uninstructed control group performed fairly well (roughly about 75%) in separating between truths and lies. In fact, this group performed the same as well as the consistency-central (both experiments) or even the evasive-A group (Experiment 2). This relatively good accuracy might be a result, at least in part, of the vast majority of uninstructed participants spontaneously using consistency or inconsistencies between the two interviews to make their judgments. Similarly, more than 30% of these participants also used cues akin to evasive answers.

In hindsight, the uninstructed group's high accuracy rate makes perfect sense, and we should have anticipated it. Prior research shows that people believe that consistency indicates truthfulness and inconsistencies indicate deception (Strömwall et al., 2004; Global Deception Research Team, 2006; Fisher et al., 2013; Vredeveldt et al., 2014). Moreover, when judging veracity, people spontaneously use between-statement consistency (Granhag and Strömwall, 2001; Strömwall and Granhag, 2005; Street and Masip, 2015). In line with previous studies, our uninstructed raters used between-statement consistency too. Because the truthful and deceptive interview pairs that we used differed significantly in terms of between-statement consistency, it was reasonable to expect that our raters would reach remarkable accuracy rates. Thus, a strength of Masip et al.'s (2016b) interview approach is that it elicits in liars a cue that people already use when judging veracity; in this way, it boosts accuracy even among uninstructed raters. However, it should be stressed that accuracy for the uninstructed control condition was higher than it was for the consistency condition; therefore, cues other than consistency (like evasive answers or others) also might have contributed to the uninstructed condition's high accuracy rate (as suggested by the data in Tables 2, 4).

One might wonder why the naïve interviewers in Masip et al.'s (2016b) experiment performed so poorly in judging the current suspects' veracity (54% overall accuracy; 71% for truths, and 40% for lies) compared to the uninstructed condition in this study. The answer is simple: None of them interviewed the same suspect twice; therefore, they could not compare the suspects' replies across the two interviews. In addition, their role as interviewers probably consumed cognitive resources that they could not employ to scrutinize the senders' responses; conversely, the raters in the current study could focus all of their attentional and cognitive resources on the veracity assessment task. Finally, Masip et al.'s (2016b) interviewers had access to nonverbal cues, which are less diagnostic of deception than verbal cues (e.g., Vrij, 2008; see also Hauch et al., 2016).

### The current procedures: the benefits of minimizing the influence of “the human factor”

Masip et al.'s (2016b) goal was to design an interview protocol to elicit *diagnostic* cues to deception that could be *easily* used to assess veracity *by any* instructed rater. This involved asking very specific questions requiring short answers—this way ambiguity would be reduced and the coding of consistency and evasive answers would be straightforward. It also involved designing detailed instructions to code and use the diagnostic cues such that any average person (rather than only the highly skilled) could be successfully instructed and could perform well in judging veracity. This was attained by designing structured materials (the transcripts and response sheets) and an almost mechanical procedure based on comparing very short and specific answers, tallying consistencies and evasive responses, and performing very simple mathematical operations. There was little room for interpretation; hence, subjectivity could hardly bias the judgments. Such an objective, structured procedure would be highly valuable in applied settings.

Indeed, all of this limited the influence of the “human factor” in the current experiments, but this was intentional. Even so, the current study goes beyond Masip et al.'s (2016b). Unlike computers, humans can (and do) commit errors, and their misguided beliefs, expectancies and stereotypes can taint their judgments. We designed materials and instructions to prevent raters from being influenced by these factors, to efficiently code and judge the veracity of all interviews, and to see whether their accuracy rates were comparable to the computerized classification rates of Masip et al. (2016b). We tested the procedures with large numbers of average raters, including police officers—a population who could benefit from the current interviewing approach—and examined the most experienced officers separately to see whether their stronger skepticism and beliefs about deception cues (e.g., Masip and Garrido, 2001; Masip et al., 2005, 2016a; Hurst and Oswald, 2012) limited the effectiveness of the instruction. Overall, accuracy rates were high across all samples, and generally comparable to the BLRAs classification rates. These findings speak in favor of our procedures.

The procedures even permitted high accuracy rates among uninstructed raters. We believe that using very specific questions contributed to their success, as did the way we presented the transcripts to them, with each suspect's response to each question during the second interview next to his/her response to the same question during the first interview (see Appendix 1 in Supplementary Material). This may have helped uninstructed raters to spontaneously compare the responses and hence to be able to correctly use consistency^12^. However, it was not the case that, because of their specific nature, these questions produced extremely telltale responses, as evidenced by the interviewers in Masip et al. (2016b) attaining only a 54% accuracy rate.

### Limitations

The current study has some limitations. First, each participant read 12 interview pairs, six truthful and six deceptive ones. Whereas, deceptive responses can vary greatly across suspects, presumably the truthful responses of different suspects were rather similar. Thus, by examining the consistency among different suspects, the raters could get some hints as to which suspects were truthful (those giving similar answers to each other) and which were deceptive (those giving different answers). This could have enhanced accuracy. Therefore, we examined the raters' use of this strategy, as well as its impact on accuracy.

Consistency/inconsistency among interviewees was amongst the cue categories that we coded for the uninstructed group. Only 12 (15%) of the 81 uninstructed participants in Experiment 1 reported having used this cue (see Table 2). Accuracy rates excluding these 12 participants (78.02% for truths, 71.50% for lies, and 74.76% overall) were very similar to those reported in Table 1 and Figure 1 for the full sample. As for Experiment 2, only one out of the 22 participants in the uninstructed condition reported having used this cue. Concerning the other conditions, the participants followed our instructions carefully, counting the number of consistencies and/or evasive answers, as reflected in the cells of the transcript and response sheets that they thoroughly filled in. Examination of these sheets shows that the raters' judgments were indeed based on the instruction cues. Finally, the current accuracy rates are not any higher than Masip et al.'s (2016b) BLRAs classification rates based solely on consistency across the two interviews and/or on evasive answers. In short, the consistency/inconsistency among interviewees was not responsible for the high accuracy rates.

Second, critics might argue that the positive findings of the current experiments merely reflect that Masip et al. (2016b) produced interviews that were very easy to be sorted into truths and lies. Indeed, the average accuracy rate across conditions (excluding the information condition) was above 75%, and the uninstructed control group performed at approximately that level. This critique is misguided. First, the interviewers, who also judged the veracity of these interviews, reached an accuracy rate of only 54% (Masip et al., 2016b), which is the same as the meta-analytical mean accuracy rate of humans judging deception from verbal and nonverbal cues (Bond and DePaulo, 2006). Also, interviewers were strongly truth biased, which is also consistent with meta-analytical findings (Bond and DePaulo, 2006). These results speak against the notion that the materials we used contained numerous telltale deception cues which were easy to spot. Second, the four experimental groups were asked to count specific cues that Masip et al. (2016b) had found to differ significantly between truthful and deceptive interviews, and that had permitted fairly high BLRAs classification rates. Raters were also given very precise instructions on how to use these cues to make their judgments. The high accuracy rates were thus not an anomaly, but the expected result. Not surprisingly, the higher (a) the effect size [calculated from Masip et al.'s (2016b) data] for the difference between lies and truths for each specific cue used by raters, and (b) the BLRAs classification rates based on these cues, the higher the accuracy rates were in the current study^13^. Third, concerning the uninstructed participants, as argued above the data show they spontaneously used the two diagnostic cues. They were able to do so because, unlike the interviewers, they could carefully compare the verbal responses from both interviews. Thus, in a sense the interviews were indeed easy to classify as truthful or deceptive, but not because they contained lots of straightforward deception cues, but because liars and truth tellers differed in terms of inconsistencies and evasive answers, which was the result of using a specific kind of interview designed to elicit precisely these deception cues to facilitate credibility assessment.

Another limitation of the current study is that because it was a proof-of-concept study to examine whether humans could reproduce the optimal outcomes of Masip et al.'s (2016b) BLRAs, the raters made their decisions using the cutoff points employed by these analyses. Had the raters been free to use cutoff points of their choice, their accuracy rates could have been different. A problem inherent to using this approach (or similar ones) in applied settings is that practitioners should first be informed about the cutoff score they should use. However, the optimal cutoff score might vary across different situations. The impact of structural features (e.g., the number of questions asked, how difficult they are, etc.) on cutoff scores can be examined in controlled laboratory experiments; however, empirically determining optimal cutoff points for specific types of real-life cases looks hardly realistic in view of (a) the large number of variables that might have an influence, and (b) the difficulty of having direct access to the ground truth in real cases (knowledge of the truth would be necessary to establish valid cutoff points).

Note that the cutoff problem affects most strategic interview approaches to detect deception, yet it has hardly (if at all) been discussed. However, the current findings for the uninstructed control group suggest there is a way out, at least for the present approach. Participants in the uninstructed condition received no instruction at all about any cutoff score to be used to make their judgments, yet their overall accuracy rate was around 75%. These findings are encouraging. Still, the question remains whether our uninstructed raters would have performed that well in a specific real life situation. Future research should explore this issue.

### Implications and future research

The current interview approach has a number of advantages, such as its brevity and the high accuracy that raters (even those who received no instruction) can reach. It could potentially be helpful in cases where the police can collect independent information about the alibi. For instance, a suspect might claim she was attending a public event on her own at the time of the crime. The police could interview a number of known attendees to collect central and peripheral information about the event to create a collection of specific questions. The suspect could then be interviewed twice and her responses transcribed for assessment.

However, some considerations are in order. First, only questions about details that almost all witnesses noticed and recall should be included in the interview. Evasive answers such as “I don't know” cannot work as a deception cue if truth tellers really do not know. Second, suspects with low IQ, highly suggestible, or with memory deficits might be at a high risk of giving evasive or inconsistent answers even if they are truthful; special care is warranted in those cases. Third, the current results suggest that if the proposed interview approach is used and the pairs of interviews are transcribed as we did, even uninstructed raters can do rather well (about 75% accuracy rate). Interestingly enough, uninstructed raters were given no specific cutoff points on any variable to make their decisions. Their high accuracy rates thus suggest a way to go around the cutoff point problem in applied settings, as discussed above. Certainly, it is still possible for the accuracy of the uninstructed group to vary from one situation to another—this is an issue to be explored by future research—but the current findings are encouraging. They also suggest an interesting novel orientation for active interview approaches to detect deception: These approaches should attempt to elicit those specific deception cues that deception judges already use spontaneously. Inconsistency is one such cue, but there are more (see Hartwig and Bond, 2011).

Fourth, a central feature of the current approach is that a second, unexpected interview needs to be conducted. However, in real cases suspects may arguably expect to be interviewed repeatedly—though it is unclear what the proportion of real suspects who have this expectation is, or what the police can do to diminish this expectation. Anticipation of the second interview might limit the use of inconsistency as a deception cue; however, note that in the current study evasive answers were more diagnostic of deception than inconsistencies, and there is no need to conduct two interviews to elicit evasive answers in liars. In fact, in the study by Masip et al. (2016b) evasive answers discriminated between liars and truth tellers both during the first and during the second interview. Thus, a useful approach could be running just one interview to elicit evasive answers. It is, however, unclear whether uninstructed raters would perform well on the basis of evasive answers alone. Uninstructed raters in the current study spontaneously used inconsistencies much more often than evasive answers to make their judgments. Also, whether the questions focused on central or peripheral details had little effect on most of the variables measured by Masip et al. (2016b); therefore, a simplified version of the current interview approach could dismiss this distinction.

Finally, it should be noted that the purpose of this interview approach is to detect deception rather than to collect abundant information from suspects. In this regard, it is similar to polygraph testing. However, the current approach is not incompatible with information gathering approaches, which could be used afterwards. Also, in contexts where there are multiple suspects, it could be used as a screening procedure before starting a real investigation focused on one or just a few suspects (i.e., those displaying many inconsistencies and/or evasive answers) to search for hard evidence, or before conducting time-consuming in-depth investigative interviews with that/those suspect(s)^14^. In this case, the optimal cutoff score problem would be ameliorated, as the proposed interview approach would be used only as a device to determine whether a suspect should be released or more and stronger evidence of his or her guilt should be sought.

Notwithstanding these arguments, we believe that using the current approach at this point would still be premature. First, it is apparent from the preceding paragraphs that there are still many questions awaiting an empirical response. Second, we believe that the current research makes more of a conceptual contribution (e.g., it provides evidence that between-statement inconsistencies *can* reveal deception, it suggests that evasive answers can have potential as a deception cue, it brings in the suggestion that strategic interview approaches to detect deception should elicit cues that deception judges already use spontaneously, it suggests there might be ways to go around the optimal cutoff point problem in applied settings, it shows the importance of minimizing the influence of “the human factor” in judging veracity, etc.), than of a practical contribution. Note that in the study by Masip et al. (2016b) correct responses discriminated better between truths and lies than did (in)consistencies and/or evasive answers. Similarly, in this experiment the informed participants performed close to perception (although in Experiment 2 the consistency-evasive group performed just at the same level). Third, as pointed out by Masip et al. (2016b), extremely high accuracy rates need to be approached with caution; replication is necessary before deriving strong advice for practitioners. Yet, recent deception detection research has yielded fairly high accuracy rates. Rather than an anomaly, these increased rates are the result of a change in orientation in lie detection research (Levine, 2015; see also Masip, 2017). Fourth, attempts to replicate and extend the current findings should ideally use more ecologically valid paradigms. Finally, once it is clear that between-statement inconsistencies *can* reveal deception, research could explore alternative procedures to elicit inconsistencies in liars in ways that can be useful in applied settings. Further research on the potential of evasive answers also seems warranted.

To conclude, we believe that the present research provides some helpful suggestions as researchers continue on the quest to develop new interview approaches to detect deception. We hope our findings and considerations will foster new research.

## Ethics statement

We conducted the current study in accordance with relevant international (American Psychological Association) and national (Código Deontológico del Psicólogo) ethics guidelines. The participants gave written informed consent, participated voluntarily, and were free to withdraw at any time.

## Author contributions

JM conceived the research, prepared the materials, analyzed the data, and drafted the manuscript. JM, CM, NS, and CH collected the data. CM also entered the data and transcribed the uninstructed condition participants' responses to the open questions. CM and NS checked the transcript sheets and the response sheets for errors. NS also coded the open responses of Experiment 1, and instructed and supervised the Experiment 2 coders. CH made the necessary arrangements to collect the data at the Police School. IB-G and II contributed to the theoretical rationale for the study and the research design. JM, IB-G, CH, and II interpreted the results. All authors critically revised and eventually approved the manuscript.

### Conflict of interest statement

The authors declare that the research was conducted in the absence of any commercial or financial relationships that could be construed as a potential conflict of interest.

## Footnotes

^1^In the study by Granhag et al. (2016), liars and truth tellers planned some actions to be performed either in a shopping mall or in a train station. They were subsequently interviewed three times with both unanticipated and anticipated questions. The authors used a more fine-grained measure of consistency than Mac Giolla and Granhag (2015); namely, they calculated the proportion of repetitions, omissions, and commissions across interviews. The only significant difference between truth tellers and liars was a difference in commissions (but not in repetitions or omissions) when answering to unanticipated questions in the train station situation (but not in the shopping mall situation). The general conclusion of the authors was that “truth tellers and liars were extremely similar with regards to between-statement consistency. This trend held across all eight situations where between-statement consistency could be measured, and with all three forms of consistency measures (omissions, commissions, and repetitions)” (p. 10).

^2^Surprisingly, it has not been until recently that the influence of delay, strength of encoding, and detail salience on truth tellers' and liars' memory has begun to be examined empirically. For recent research on these topics, see Roos af Hjelmsäter et al. (2014), Harvey et al. (2017a,b), and Sakrisvold et al. (2017).

^3^Of course, there are certain circumstances where lying does not involve more cognitive load than truth telling (see Burgoon, 2015; Sporer, 2016; Blandón-Gitlin et al., 2017).

^4^Masip et al. (2016b) explained this unexpected finding in terms of the *forced confabulation effect*: When people generate answers to *unanswerable* questions, they might incorporate the confabulated responses in their memory network and provide the same answer during subsequent interviews (Pezdek et al., 2007). It is also possible that guilty participants being asked a peripheral question to which they ignored the answer experienced the situation as unexpected, salient, and thrilling, which might have facilitated memory encoding of the circumstance (including the response given) during the first interview, as well as memory retrieval during the second interview (see Bradley et al., 1992).

^5^Although in the study by Masip et al. (2016b) the difference between guilty and innocent suspects in terms of evasive answers was larger in replying to peripheral than to central questions, the difference was significant in replying to both kinds of questions. In addition, the overall effect size for the difference between guilty and innocent suspects across both question types was very large (*d* = 2.11). Therefore, we decided to include in the current study a group instructed to focus on evasive answers overall rather than on evasive answers in responding to peripheral questions only.

^6^By multiplying the sum times 6.25 the result was transformed from a 0-to-16 scale to a 0-to-100 scale; in other words, the *percentage* of consistent answers was obtained. The calculation is based on a simple mathematical “rule of three”: If 16 ⇒ 100% and Sum ⇒ *X*, then *X* = Sum × (100/16) = Sum × 6.25.

^7^For example, the BLRA cut-off point for the consistency and evasive answers combined variable was 73% (Masip et al., 2016b). Thus, in the current study we instructed participants in the consistency-evasive condition to judge those suspects for whom the final percentage was lower than 73% as deceptive, and those for whom the final percentage was 73% or higher as truthful.

^8^The instructions, the correct answer sheet, and the response sheets are available (in Spanish) from the first author on request.

^9^For each group, the eight different versions of the transcript booklets [4 Sets × 2 Interview Orderings (direct and reverse)] were interspersed; thus, the number of participants receiving each version was about the same.

^10^We coded as “undefined (in)consistency” those responses in which the raters mentioned consistency or inconsistency without indicating whether they referred to (in)consistencies across the two interviews, within the same interview (i.e., the same suspect giving (in)consistent answers to different questions in the interview), or among different interviewees [i.e., different suspects giving (in)consistent answers when asked the same (set of) questions].

^11^Note, however, that in Experiment 2 the participants rated only a subset of 12 transcripts. Therefore, the Experiment 2 findings can be less representative of the actual state of affairs than those of Experiment 1.

^12^We are grateful to Dr. Joanna Ulatowska (Maria Grzegorzewska University, Warsaw, Poland) for this observation.

^13^Cohen's *d*s were 1.19 for consistency, 1.41 for consistency for central questions, 2.11 for evasive answers, and 2.59 for the combined consistency and evasive answers variable. The BLRAs classification rates were 69, 73, 87, and 94%, respectively.

^14^We are grateful to Antonio Domínguez (Behavior & Law Foundation, Madrid, Spain) for this suggestion.
