# Supplementary material for: Learning to Detect Deception from Evasive Answers and Inconsistencies across Repeated Interviews: A Study with Lay Respondents and Police Officers
Source: Front Psychol. 2018 Jan 4;8:2207. doi: 10.3389/fpsyg.2017.02207 (PMC5758596; doi:10.3389/fpsyg.2017.02207)
Supplement: Supplementary file 1 [file Appendix.docx]

# Appendix 1: Transcript Page for the Consistency Condition Corresponding to Suspect 7 in Set 3-D

| 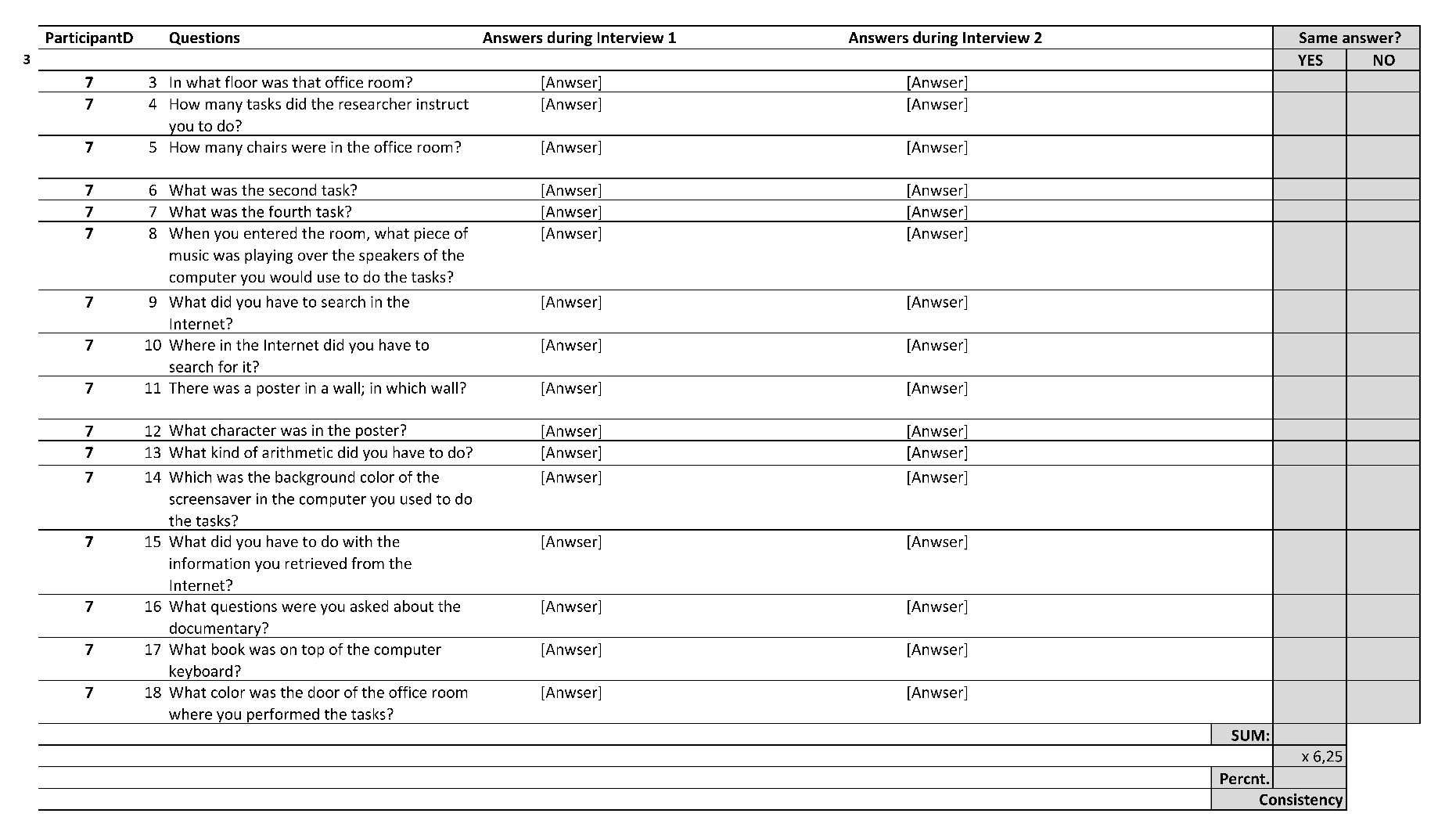 |
| --- |
| *Notes.* The number on the top left side (“3”) is the set number, while the letter “D” after “Participant” stands for “direct” order; these codes were for the researchers only. Questions 1 and 2 were “Did you steal a wallet in Seminar Room 126? (All suspects replied “No”), and “Then you have done some tasks in an office room with a researcher?” (All suspects replied “Yes”); therefore, only 16 questions (questions 3 through 18) were relevant. For confidentiality reasons, no actual participant’s answers are shown on this table. |

# Appendix 2: Analyses with the Corrected Data

## Experiment 1

### Veracity x Condition ANOVA on accuracy

- Veracity main effect: *F* (1, 469) = 4.73, *p* = .030, η_p_^2^ = .010.
- Condition main effect: *F* (5, 469) = 128.67, *p* < .001, η_p_^2^ = .578.
- Veracity x Condition interaction: *F* (5, 469) = 8.85, *p* < .001, η_p_^2^ = .086.
- No other effect was significant.

Appendix Table 1

| *Mean Accuracy Rates (Standard Deviations) for the Different Conditions in Experiment 1 (Corrected Data)* | | | | | | | |
| --- | --- | --- | --- | --- | --- | --- | --- |
| Veracity | Consistency* | Consistency-Central* | Uninstructed* | Evasive-A | Consistency-Evasive | Information | Across Conditions* |
| Lies | 69.41^a^ (16.98) | 66.40^a^ (18.77) | 72.84^a^ (19.62) | 85.28^b^ (10.82) | 88.33^b^ (15.40) | 99.24^c^ (3.49) | 80.65 (19.12) |
| Truths | 59.66^a^ (20.33) | 80.18^b^ (16.01) | 79.01^b^ (17.03) | 88.96^c^ (14.71) | 88.07^c^ (16.20) | 99.43^d^ (3.04) | 82.82 (19.72) |
| *Overall* | *64.54^a^ (9.89)* | *73.29^b^ (10.43)* | *75.93^b^ (15.48)* | *87.12^c^ (5.37)* | *88.20^c^ (11.15)* | *99.34^d^ (2.27)* | *81.74 (15.29)* |

*Note.* Scheffé tests were conducted for pairwise comparisons. Within each row, means with different superscripts differ significantly from each other. For those columns with an asterisk in the column heading the difference in accuracy between judging truths and lies was significant.

## Experiment 2

### All Officers: Veracity x Condition ANOVA on Accuracy

- Veracity main effect: *F* (1, 136) = 1.66, *p* = .199, η_p_^2^ = .012 (not significant).
- Condition main effect: *F* (5, 136) = 48.76, *p* < .001, η_p_^2^ = .642.
- Veracity x Condition interaction: *F* (5, 136) = 7.04, *p* < .001, η_p_^2^ = .206.
- No other effect was significant.

| Appendix Table 2  *Officers’ Mean Accuracy Rates (Standard Deviations) for the Different Conditions in Experiment 2 (Corrected Data)* | | | | | | | |
| --- | --- | --- | --- | --- | --- | --- | --- |
| Veracity | Consistency* | Consistency-Central | Uninstructed* | Evasive-A* | Consistency-Evasive | Information | Across Conditions |
| Lies | 68.75^a,b^ (13.29) | 71.73^a,b^ (15.16) | 66.67^a^ (25.72) | 80.00^b,c^ (14.14) | 92.27^c,d^ (10.10) | 100.00^d^ (0.00) | 79.72 (19.16) |
| Truths | 52.50^a^ (21.72) | 69.73^b^ (15.33) | 87.12^c^ (17.01) | 92.82^c^ (9.78) | 95.45^c^ (17.95) | 97.10^c^ (10.84) | 82.16 (22.57) |
| *Overall* | *60.63^a^ (10.07)* | *70.73^b^ (10.00)* | *76.89^b,c^ (14.30)* | *86.41^c,d^ (8.92)* | *93.86^d,e^ (9.71)* | *98.55^e^ (5.42)* | *80.94 (16.44)* |

*Note.* Scheffé tests were conducted for pairwise comparisons. Within each row, means with different superscripts differ significantly from each other. For those columns with an asterisk in the column heading the difference in accuracy between judging truths and lies was significant.

### Experienced Officers Only: Veracity x Condition ANOVA on Accuracy

- Condition main effect: *F* (5, 87) = 26.76, *p* < .001, η_p_^2^ = .606.
- Veracity x Condition interaction: *F* (5, 87) = 4.40, *p* = .001, η_p_^2^ = .202 .
- No other effect was significant.

| Appendix Table 3  *Experienced Officers’ Mean Accuracy Rates (Standard Deviations) for the Different Conditions in Experiment 2 (Corrected Data)* | | | | | | | | |
| --- | --- | --- | --- | --- | --- | --- | --- | --- |
| Veracity | Consistency* | Consistency-Central | Uninstructed* | Evasive-A | Consistency-Evasive | Information | Across Conditions |  |
| Lies | 70.83^a,b^ (14.43) | 72.50^a,b^ (17.32) | 62.50^a^ (25.46) | 80.20^b,c^ (13.46) | 90.98^c,d^ (10.59) | 100.00^d^ (0.00) | 79.78 (19.80) |  |
| Truths | 53.61^a^ (23.59) | 66.25^a^ (17.29) | 84.38^b^ (18.73) | 90.98^b^ (10.59) | 94.12^b^ (20.36) | 98.89^b^ (4.30) | 82.62 (22.46) |  |
| *Overall* | *62.22^a^ (12.25)* | *69.38^a^ (11.35)* | *73.44^a,b^ (13.68)* | *85.59^b,c^ (9.75)* | *92.55^c,d^ (10.59)* | *99.44^d^ (2.15)* | *81.20 (16.42)* |  |

*Note.* Scheffé tests were conducted for pairwise comparisons. Within each row, means with different superscripts differ significantly from each other. For those columns with an asterisk in the column heading the difference in accuracy between judging truths and lies was significant.

### Officers and Students Who Had Rated the Same Set as Officers: Sample x Veracity x Condition ANOVA on Accuracy

- Sample main effect: *F* (1, 246) = 1.36, *p* = .244, η_p_^2^ = .006 (not significant).
- Condition main effect: *F* (5, 246) = 104.50, *p* < .001, η_p_^2^ = .680.
- Veracity x Condition interaction: *F* (5, 246) = 10.32, *p* < .001, η_p_^2^ = .173.
- No other effect was significant.
